# Mixomics analysis of *Bacillus subtilis*: effect of oxygen availability on riboflavin production

**DOI:** 10.1186/s12934-017-0764-z

**Published:** 2017-09-12

**Authors:** Junlang Hu, Pan Lei, Ali Mohsin, Xiaoyun Liu, Mingzhi Huang, Liang Li, Jianhua Hu, Haifeng Hang, Yingping Zhuang, Meijin Guo

**Affiliations:** 1 State Key Laboratory of Bioreactor Engineering, East China University of Science and Technology, 130 Meilong Rd., P.O. box 329#, Shanghai, 200237 People’s Republic of China; 2 Shanghai Acebright Pharmaceuticals Group Co., Ltd, Shanghai, 201203 People’s Republic of China

**Keywords:** Riboflavin, *Bacillus subtilis*, Oxygen availability, ^13^C metabolic flux analysis, Metabolomics, Transcriptomics

## Abstract

**Background:**

Riboflavin, an intermediate of primary metabolism, is one kind of important food additive with high economic value. The microbial cell factory *Bacillus subtilis* has already been proven to possess significant importance for the food industry and have become one of the most widely used riboflavin-producing strains. In the practical fermentation processes, a sharp decrease in riboflavin production is encountered along with a decrease in the dissolved oxygen (DO) tension. Influence of this oxygen availability on riboflavin biosynthesis through carbon central metabolic pathways in *B. subtilis* is unknown so far. Therefore the unveiled effective metabolic pathways were still an unaccomplished task till present research work.

**Results:**

In this paper, the microscopic regulation mechanisms of *B. subtilis* grown under different dissolved oxygen tensions were studied by integrating ^13^C metabolic flux analysis, metabolomics and transcriptomics. It was revealed that the glucose metabolic flux through pentose phosphate (PP) pathway was lower as being confirmed by smaller pool sizes of metabolites in PP pathway and lower expression amount of *ykgB* at transcriptional level. The latter encodes 6-phosphogluconolactonase (6-PGL) under low DO tension. In response to low DO tension in broth, the glucose metabolic flux through Embden–Meyerhof–Parnas (EMP) pathway was higher and the gene, *alsS*, encoding for acetolactate synthase was significantly activated that may result due to lower ATP concentration and higher NADH/NAD^+^ ratio. Moreover, ResE, a membrane-anchored protein that is capable of oxygen regulated phosphorylase activity, and ResD, a regulatory protein that can be phosphorylated and dephosphorylated by ResE, were considered as DO tension sensor and transcriptional regulator respectively.

**Conclusions:**

This study shows that integration of transcriptomics, ^13^C metabolic flux analysis and metabolomics analysis provides a comprehensive understanding of biosynthesized riboflavin’s regulatory mechanisms in *B. subtilis* grown under different dissolved oxygen tension conditions. The two-component system, ResD–ResE, was considered as the signal receiver of DO tension and gene regulator that led to differences between biomass and riboflavin production after triggering the shifts in gene expression, metabolic flux distributions and metabolite pool sizes.

**Electronic supplementary material:**

The online version of this article (doi:10.1186/s12934-017-0764-z) contains supplementary material, which is available to authorized users.

## Background

Riboflavin (vitamin B_2_) is a water-soluble vitamin that is produced by microorganisms and plants. It is a precursor of flavin adenine dinucleotide (FAD) and flavin mononucleotide (FMN), which are cofactors involved in several important metabolic reactions. Nutritional deficiency of riboflavin can cause a variety of fundamental and clinical abnormalities from mitochondrial dysfunction and hemolytic anemia to growth retardation and neurological disorders [1]. Low blood counts, dizziness, hair loss and poor digestion can also be attributed as symptoms to the riboflavin deficiency. It can also be used in the treatment of neonatal jaundice, corneal disorder [2] and migraine [1]. Beside these nutritive and medicinal values, riboflavin as a supplement is essentially required in animal raising [3].

As a model organism, *Bacillus subtilis* has played an important role in the medicinal industry [4] and possess the potential to produce riboflavin. In order to have commercial scale production of riboflavin by *B. subtilis*, many researches have focused on the establishment of productive strains [5, 6] and optimal fermentation strategies [7, 8]. In industrial scale fermentation processes, we found that the yield of riboflavin decreased sharply from 1.08 to 0.59 g riboflavin/g glucose while the dissolved oxygen (DO) tension was below 30% of saturated concentration in water (37 °C, 0.15 MPa). However, to maintain DO tension above 30% during the later phase of fermentation is difficult and highly energy-consumptive especially for production level of industrial scale. Hence, enhanced production at lower DO tension can economize the overall process. As we already know, oxygen is an essential substrate in most of aerobic organisms’ metabolic processes because of its significance as electron receptor. Oxygen deficiency may lead to low efficiency of NADH and NADPH oxidation that are the essential reactions to produce ATP and low flux through tricarboxylic acid (TCA) cycle [9]. It is reasonable to hypothesize that a low energy conversion rate can affect the cell multiplication that leads to lower riboflavin production. In past decade, the development of OMICS and high-throughput technology enabled the further study on cellular molecular regulatory mechanism during different biological processes. For examples, the whole genome sequence of *B. subtilis* 168 was completed in 1997 [4], since then gene re-sequencing has been used to find out the distinction of different *B. subtilis* strains based on the genomic DNA data. Global gene expression and metabolomics analysis were used to identify the response to different oxygen availability in the riboflavin fermentation. Furthermore, ^13^C metabolic flux analysis (MFA) was also employed to reveal the metabolic flux shift between these two conditions. By integrating these omics data and analysis of ^13^C MFA, it is possible to get a comprehensive understanding of regulatory mechanisms of riboflavin-producing strain from gene expression and metabolic levels.

In the previous studies, many researchers developed the fermentation process of riboflavin by *B. subtilis*, but the majority of them focused on the macroscopic process behavior [10] or gene manipulation for strain development using metabolic engineering technology [11]. However, it was the first time that Sauer et al. on quantifying the metabolic fluxes of a wild-type strain and an industrial riboflavin-producing *B. subtilis*, found that the metabolic flux through PP pathway was growth rate-dependent and higher in the riboflavin-producing strain [5]. Then Shi et al. integrated a comparative transcriptome profiling and metabolic pool analysis to explore the differences between a riboflavin-producing and the wild-type strain, and revealed that the lower expression of PurR-regulated genes was the bottleneck for increasing riboflavin production in their riboflavin-producing strain [12]. These Pur-regulated genes are one set that are involved in purine metabolism and glycine biosynthesis. The expression of these genes can be inhibited by PurR and may lead to lower riboflavin production. To the best of our knowledge, no one ever has revealed the mechanism of riboflavin’s biosynthesis by *B. subtilis* under the influence of oxygen availability. Fast-growing aerobic microorganisms like *B. subtilis* require large amount of oxygen for oxidation of NAD(P)H or FADH_2_ to generate enough ATP for the growth and targeted bio-product formation [13]. Despite its occurrence during fermentation, 12 mol ATP per mol of synthesized product is required that makes this process as highly oxygen consuming. Therefore, this work aims to bring insights into the possible regulatory roles of oxygen in riboflavin biosynthesis by an industrial strain *B. subtilis* cultivated under different DO tension conditions using integrated ^13^C MFA, metabolomics and transcriptomics analysis.

## Methods

### Strain and cultivations

*Bacillus subtilis* was stored at Aceibright Company (Shanghai, China), which was mutated by chemicals like NTG and physical method like UV and ^60^Co radiation, and genomic DNA was shuffled for many rounds [14].

Fermentation was carried out as follows: a 100 μL of seed vial, stored in 50% glycerol at −80 °C, it was spread on a Petri dish containing solid medium, and cultured at 37 °C for 24 h. Then, a single colony grown on Petri dish was picked into a 500 mL shaking flask containing 50 mL seed medium, and incubated in a rotary shaker at 37 °C and 220 rpm for 24 h.

Based on the fermentation process, sudden decrease of riboflavin production was observed as DO concentration decreased below 20%. So we considered 20% of saturated DO concentration under 37 °C, 0.15 MPa as critical oxygen concentration and set high DO level above 30% and low DO level in range of 0–10%. Batch cultures in a 5 L stirred bioreactor (FUS-5L, Shanghai, China) were conducted with 2.0% of inoculum for initial 3.0 L working volume at 37 °C. Aeration is kept at 2.0 VVM and process pH was controlled at 7.2 with 2.0 M sodium hydroxide. During the fermentation, agitation speed (maximum 800 rpm) was adopted to maintain dissolved oxygen (DO) tension above 30% of air saturation in the first 24 h, then shift to the high DO level of 35.0 ± 5.0% (Batch H) or low DO level of below 10% (Batch L) till the end of fermentation, respectively. The cultivations for each procedure (high DO level or low DO level) were performed in three independent cultivation replicates.

The solid medium contained (g/L) peptone 15, yeast extract 7.5, NaCl 6.5, maltose 25, agar 20, erythromycin 0.01 and chloramphenicol 0.01. The seed medium contained (g/L) glucose·H_2_O 10, corn steep liquor 15, molasses 15, yeast extra 7.5, MgSO_4_·2H_2_O 0.5, (NH_4_)_2_SO_4_ 7.5. The fermentation medium contained (g/L) glucose·H_2_O 20, K_2_HPO_4_·3H_2_O 15, KH_2_PO_4_ 6, (NH_4_)_2_SO_4_ 8, MgSO_4_·2H_2_O 0.2, erythromycin 0.01 and chloramphenicol 0.01. Besides, 10 mL/L trace element buffer that contained (g/L) CaCl_2_·2H_2_O 0.55, FeCl_3_ 1.0, MnCl_2_·4H_2_O 0.1, ZnCl_2_ 0.17, CuCl_2_·2H_2_O 0.043, CoCl_2_·6H_2_O 0.06, Na_2_MoO_4_ 0.06 was added into the fermentation medium.

### Quantification of biomass, riboflavin and extracellular glucose

For biomass weight, 5 mL culture was taken and filtered through pre-dried and pre-weighed filter paper. To remove solutes, the cells were rinsed three times with deionized water. Then the wet filters with the biomass were put in the 80 °C oven and dried for 24 h. The dried filter was re-weighed immediately.

Regarding riboflavin determination, 0.05% sodium hydroxide solution was used to dissolve the riboflavin in the broth. After appropriate dilution, absorbance of sample was detected by spectrophotometer (A_444_).

A glucose analyzing Kit (Shanghai Kexin Biotechnology Institute, China) was used for quantitative analysis for glucose concentration. After centrifugation (4000 rpm, 10 min) and appropriate dilution of samples, they were mixed with reagents provided by the Kit and maintained 37 °C for 20 min before determination by spectrophotometer (A_510_).

### Analysis of extracellular organic acids

The concentrations of extracellular organic acids in the culture were analyzed using a high-performance liquid chromatography (HPLC) system. An ion-exclusion column (Hi-Plex H, Agilent) was eluted at 50 °C with 10 mM H_2_SO_4_ at a flow rate of 0.5 mL/min and connected with an absorbance detector spectrophotometer at 210 nm. 1 mL broth was centrifuged at 12,000 rpm, 4 °C for 5 min and the supernatant can be analyzed after appropriate dilution and filtration by filter membrane of diameter 0.22 μm.

### Sampling and quantification of intracellular metabolites concentration

Because of the massive leaking of metabolites into the quenching reagent in our previous experiments and other studies [15], we adapted a rapid filtration protocol for sampling instead of quenching. 1 mL broth was filtered through a vacuum filtration device, then the filter cake was washed by precooled 2.3% (m/m) sodium chloride solution. This step was performed as soon as possible. Subsequently, the filter cake with filter paper was transferred rapidly to 10 mL 75% (v/v) ethanol solution and the extraction continued for 4 min at 95 °C. To accurately determine metabolite’s concentration, 30 μL of ^13^C-labeled cell extract was added to every sample as internal standard for isotope dilution mass spectrometry (IDMS) before extraction. The supernatant was evaporated through a rotary evaporator at −101 kPa (relative pressure) and then stored at −80 °C before determination by UPLC-MS/MS (Thermo Fisher Scientific Corporation, Waltham, USA).

### ^13^C-glucose labeling experiments

^13^C-labeling experiments were conducted in a 250 mL bioreactor (NCBio, Shanghai, China) with the working volume of 150 mL using glucose as sole carbon source. The labeled glucose contained 30% natural glucose, 20% [1-^13^C]-glucose and 50% [U-^13^C]-glucose. The labeling experiments were performed in two independent cultivation bioreactors.

### Sampling and extraction of intracellular free amino acids

5 mL broth was taken during the ^13^C-labeling experiments for analysis of mass isotopomer distributions (MID) of intracellular free amino acids and rapidly mixed with 15 mL pre-cooled quenching reagent (60% methanol, −40 °C). The cells were washed twice with deionized water after 5 min centrifugation at 4 °C, 7500 rpm. Then the intracellular free amino acids were extracted with 75% (v/v) ethanol solution at 95 °C for 4 min. The supernatant was evaporated through a rotary evaporator at −101 kPa (relative pressure) before determination by gas chromatography–mass spectrometry (GC–MS).

### ^13^C-labeled metabolic flux analysis

A stoichiometric model of *B. subtilis* with compartments and reversible reactions was constructed based on the models as previously described [16]. To determine the goodness-of-fit, the ^13^C-metabolic flux analysis (MFA) fitting results were subjected to a χ^2^-statistical test. The estimated fluxes were considered acceptable only when the obtained minimal weighted residual was below the χ^2^ at a 95% confidence level. At convergence, accurate 68 and 95% confidence intervals were computed for all estimated fluxes by evaluating the sensitivity of the minimized SSR to flux variations.

### Transcriptome analysis

To study the gene expressing distinctions between the high DO level mode and the low DO level mode, during whole fermentation process, three sampling points were set in each culture mode. The supernatant was removed after centrifugation at 4 °C, 4000 rpm for 5 min. Then the cell pellets were stored at −80 °C till analysis. The extraction of mRNA, reverse transcription and sequencing were done by Shanghai Majorbio Bio-Pharm Technology Co., Ltd (Shanghai, China).

### Real-time PCR

A 5 mL broth was taken and the supernatant was removed by centrifugation at 4000 rpm for 5 min at 4 °C. Then cDNA samples were prepared by RNA extraction and reverse transcription according to RNAiso Plus (Takara Biotechnology Co., Ltd, Dalian, China) and PrimeScript™RT reagent Kit (Takara Biotechnology Co., Ltd, Dalian, China), separately. For real-time PCR, 2 μL cDNA sample, 0.6 μL forward primer, 0.6 μL reverse primer, 6.8 μL sterile deionized water and 10 μL SYBR premix dimer eraser (2×) were mixed for one reaction and the reaction was performed in a CFX96 Touch™ Real-Time PCR Detection System (Bio-Rad, CA, USA). Primers for each gene were listed in Table 1.

**Table 1:** Primer sequences used for real-time PCR

Gene	Primer sequence
16S rRNA	(F) 5′-GGGGAGCGAACAGGATTAGA-3′ (R) 5′-AGCACTAAGGGGCGGAAAC-3′
*sucD*	(F) 5′-CGATTATGGAAGCGGTAGA-3′ (R) 5′-ATGACACCAGGACAGTTCG-3′
*sdhA*	(F) 5′-AGATACGAAGTTGCGGGTC-3′ (R) 5′-GCCATAATGACAGCGTCTG-3′
*mdh*	(F) 5′-AAAGAGCGTGTAATCGGC-3′ (R) 5′-CGCCAGCATAAGAATAACG-3′
*ykgB*	(F) 5′-CAGTATCGCCGTATTTGAAG-3′ (R) 5′-AAACAAGACAAGGTTGCCTG-3′

## Results

### Fermentation performance of *B. subtilis* cultivated under different DO tension conditions

**Fig. 1:**
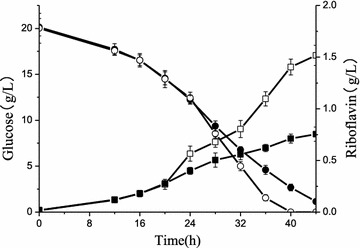
Profiling of glucose concentration and riboflavin production in two culture modes. Hollow icons represent Batch H (high DO tension), solid icons represent Batch L (low DO tension), circle represents glucose concentration, and square represents riboflavin production levels

**Fig. 2:**
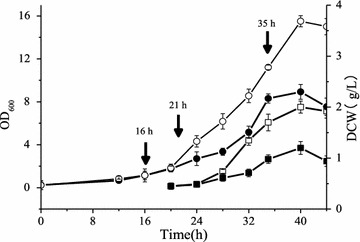
Profiling of cell optical density and dry cell weight in two batch culture. Hollow icons represent Batch H (high DO tension), solid icons represent Batch L (low DO tension), circle represents optical density, and square represents dry cell weight. Three arrows represent three sampling time points in transcriptomics and the third arrow also represents the sampling time point of ^13^C MFA and metabolomics

**Fig. 3:**
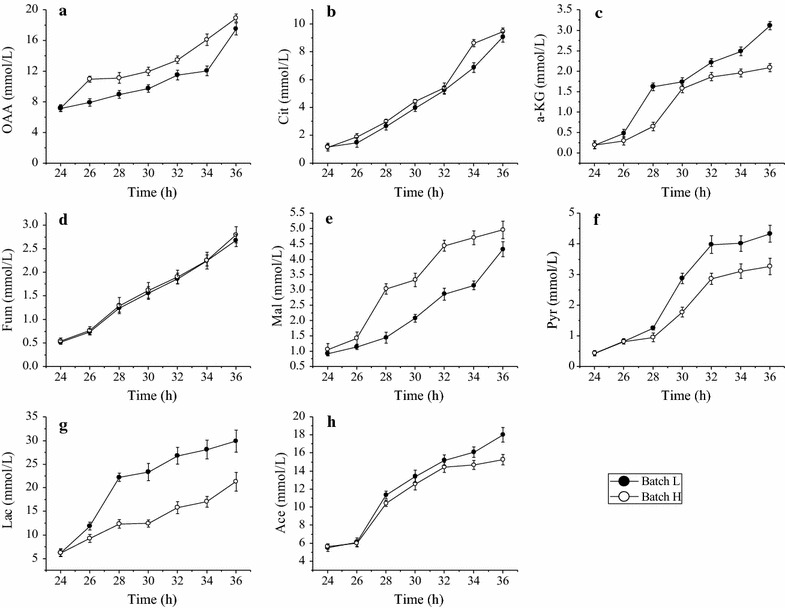
Extracellular pool sizes of extracellular organic acid determined in two batches. **a** Oxaloacetic acid, **b** citrate, **c** α-ketoglutarate, **d** fumarate, **e** malate, **f** pyruvate, **g** lactate and **h** acetate

The performance of *B. subtilis* cells cultivated under two different DO tension conditions was characterized. At first 24 h, it showed a similar performance in both batches because of the controlled DO levels above 30%. After 24 h, the DO tension was adjusted and phenotypical performance of these two bioprocesses became diverged (Figs. 1, 2). The glucose was consumed faster under high DO and depleted at about 40 h, while there was about 1 g/L glucose left in broth at 44 h in Batch L mode. Compared with the results obtained in Batch H, the pool sizes of extracellular organic acids like lactate and acetate showed a similar trend with pyruvate that increased along with the fermentation and were larger in Batch L (Fig. 3). These results were in opposite direction with the pool sizes of organic acids that involved in TCA cycle which were smaller in *B. subtilis* cells grown under low DO tension condition (Fig. 3). These smaller pool sizes of OAA and Mal indicated its lower production or maybe larger consumption into a new TCA cycle in Batch L. This can be revealed by metabolic flux analysis. Importantly, the level of riboflavin accumulated in broth increased dramatically after 24 h with higher specific growth rate (*μ* = 0.092 h^−1^) in Batch H (Table 2). It infers that oxygen availability plays key roles in riboflavin biosynthesis by *B. subtilis*. In order to elucidate regulatory mechanism of oxygen affecting riboflavin production, mixomics analysis for *B. subtilis* was carried out in the following experiments.

**Table 2:** Growth kinetic parameters of *B. subtilis* in two modes

	Batch L	Batch H
qGlc (mmol/gDCW/h)	5.66	5.32
qRib (g/gDCW/h)	0.038	0.054
*μ* (h^−1^)	0.035	0.092
Y_X/S_ (gDCW/gGlc)	0.034	0.096
Y_P/X_ (gRib/gGlc)	1.08	0.59
RQ	0.98	0.95
Rib (36 h) (g/L)	0.62	1.09
CER (mmol/gDCW/h)	4.47	9.52
OUR (mmol/gDCW/h)	4.56	10.02

*Glc* glucose, *Rib* riboflavin, *DCW* dry cell weight, *RQ* respiratory quotient, *CER* carbon dioxide expiration rate, *OUR* oxygen uptake rate

### ^13^C metabolic flux analysis

**Fig. 4:**
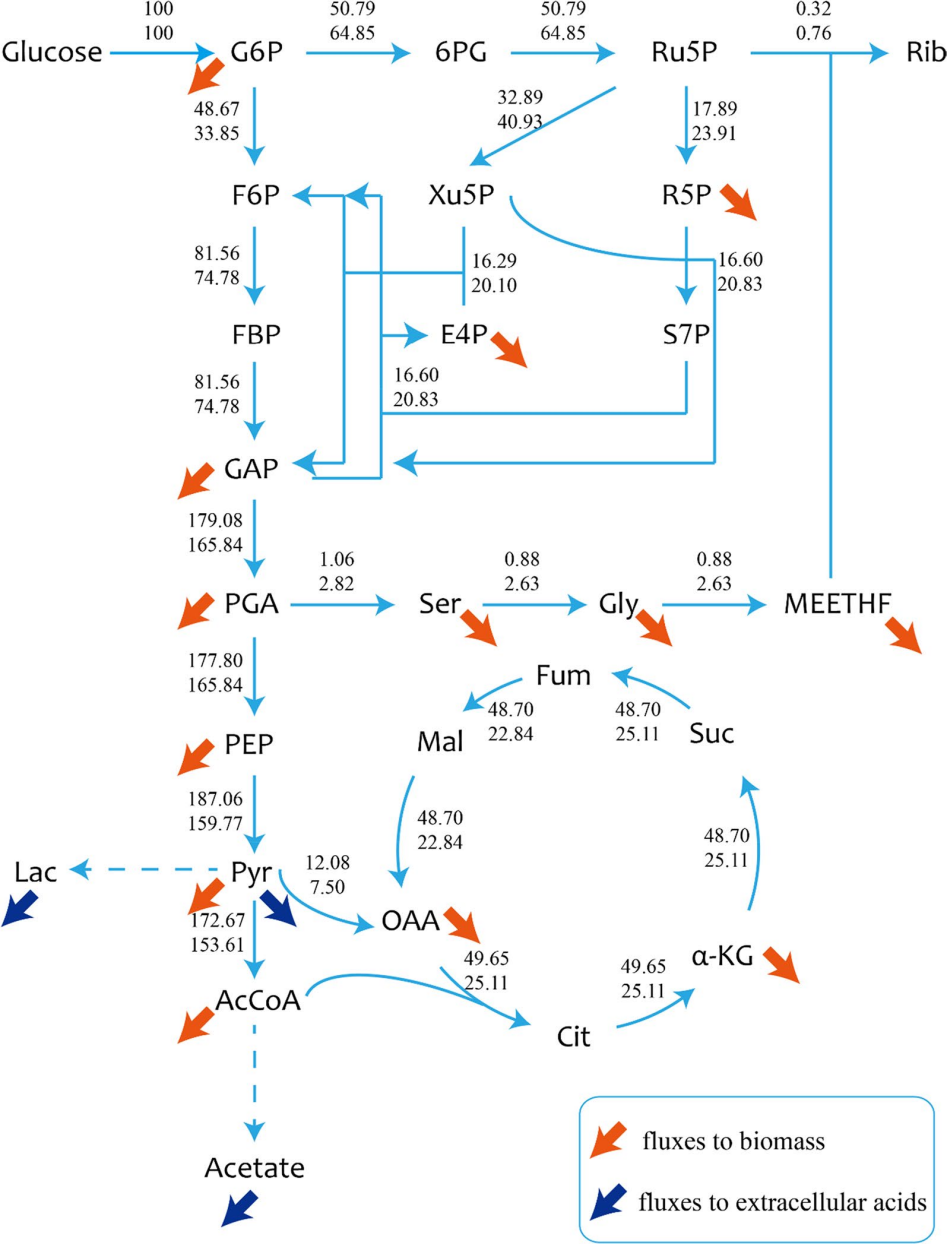
Related carbon flux distributions under different dissolved oxygen concentrations. Batch L (top), Batch H (bottom). All fluxes are normalized to the specific glucose consumption rate. G6P represents glucose 6-phosphate, 6PG represents 6-phosphogluconate, Ru5P represents ribulose 5-phosphate, Rib represents riboflavin, F6P represents fructose 6-phosphate, Xu5P represents xylulose 5-phosphate, R5P represents ribose 5-phosphate, FBP represents fructose 1,6-diphosphate, E4P represents erythrose 4-phosphate, S7P represents sedoheptulose 7-phosphate, GAP represents 3-phosphoglycerate, PGA represents 3-phosphoglycerate, Ser represents serine, Gly represents glycine, PEP represents phosphoenolpyruvate, Pyr represents pyruvate, Lac represents lactate, AcCoA represents acetyl-CoA, Cit represents citrate, α-KG represents α-ketoglutarate, Suc represents succinate, Fum represents fumarate, Mal represents malate, OAA represents oxaloacetic acid

INCA (Isotopomer Network Compartmental Analysis) software package (v1.5) was used to determine metabolic fluxes based on kinetic parameters (Table 3), the isotopic composition of glucose in broth and mass isotopomer distributions of intracellular free amino acids (Additional file 1). The estimated metabolic fluxes were thought to be statistically acceptable when the obtained minimal weighted residual from the parameter estimations was below the cut-off value determined from the χ^2^ at a 95% confidence level. The impacts of low DO level on *B. subtilis* producing riboflavin were then inferred from the estimated metabolic flux distributions (Fig. 4).

**Table 3:** Carbon recovery during ^13^C-labeling experiments under different dissolved oxygen concentrations

	Batch L	Batch H
Glucose (mmolC/gDCW/h)	33.96	31.92
Biomass (mmolC/gDCW/h)	1.43	3.77
CER (mmolC/gDCW/h)	4.47	9.52
Riboflavin (mmolC/gDCW/h)	1.70	2.38
By-products (mmolC/gDCW/h)	26.01	15.79
Carbon recovery (%)	98.94	98.56

Obvious differences were found in the central carbon fluxes between two batches. The relative flux (fluxes normalized to glucose uptake rate) through PP pathway was higher in Batch H (64.85%) than that in Batch L (50.79%). PP pathway offered precursors for nucleotide and amino acid biosynthesis which are essential for the cell growth [17]. Furthermore, Ru-5P is a precursor of riboflavin, also generated by PP pathway. This remarkable flux reduction through PP pathway may be responsible for the lower specific growth rate and riboflavin production rate (Table 2) in Batch L. Compared to Batch H, the fluxes through EMP pathway and TCA cycle were much higher in Batch L. The increased flux through EMP pathway (48.67% vs 33.85%) was predictable because cells would need ATP generated by substrate phosphorylation through EMP pathway under low oxygen availability environment. However, the higher flux through TCA cycle (12.08% vs 7.50%) was not expected. To explain this phenomenon, other analyses were also needed.

**Fig. 5:**
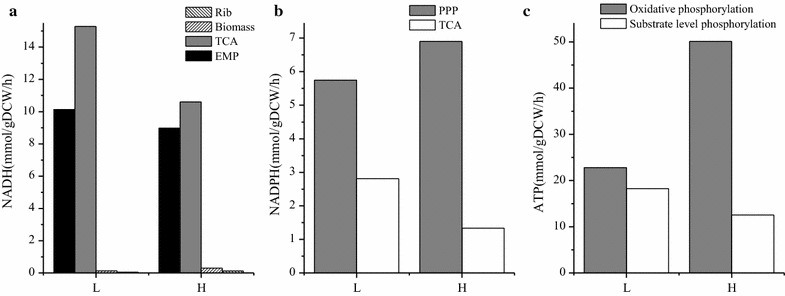
The regeneration rates of **a** NADH, **b** NADPH and **c** ATP under different DO levels

Based on the metabolic flux distribution, the generation rate of NAD(P)H from different pathways and ATP production by substrate level phosphorylation can be calculated (Fig. 5). By setting the oxidative phosphorylation rate of NADH in the respiratory chain to 2.5, corresponding to the generation of 2.5 ATP molecules per O, the ATP generation rate of oxidative phosphorylation can be calculated (Fig. 5c) based on the data of OUR (Table 2). There are two main pathways generating NADH, including EMP pathway and TCA cycle. Higher NADH generation rate was calculated in Batch L than that in Batch H because of higher flux through EMP pathway and TCA cycle (Fig. 5). When it comes to NADPH, the total producing rate was similar in two batches (Fig. 5). The specific producing rate of ATP can be calculated based on oxygen uptake rate. The oxidative phosphorylation rate was set to 2.5 (2.5 mol ATP/1 mol O) and the producing rate of ATP under different DO levels were calculated (Fig. 5c) based on OUR (Table 2). More than two times of ATP was generated by oxidative phosphorylation in Batch H (50.1 mmol/gDCW/h) than that in Batch L (22.8 mmol/gDCW/h). Slight reduction was observed in the producing rate of ATP through substrate level phosphorylation in Batch H (12.54 mmol/gDCW/h) than in Batch L (18.27 mmol/gDCW/h) because of lower flux through EMP in Batch H.

It can be speculated based on MFA that in Batch L, lower flux through PP pathway led to lower specific growth rate and riboflavin production rate. Higher flux through EMP pathway and TCA cycle may cause higher NADH generating rate but lower ATP producing rate because of deficiency of electron receptors like oxygen. These results can be explained by following metabolomics and transcriptomics analysis.

### Metabolic pools in central carbon metabolism

**Fig. 6:**
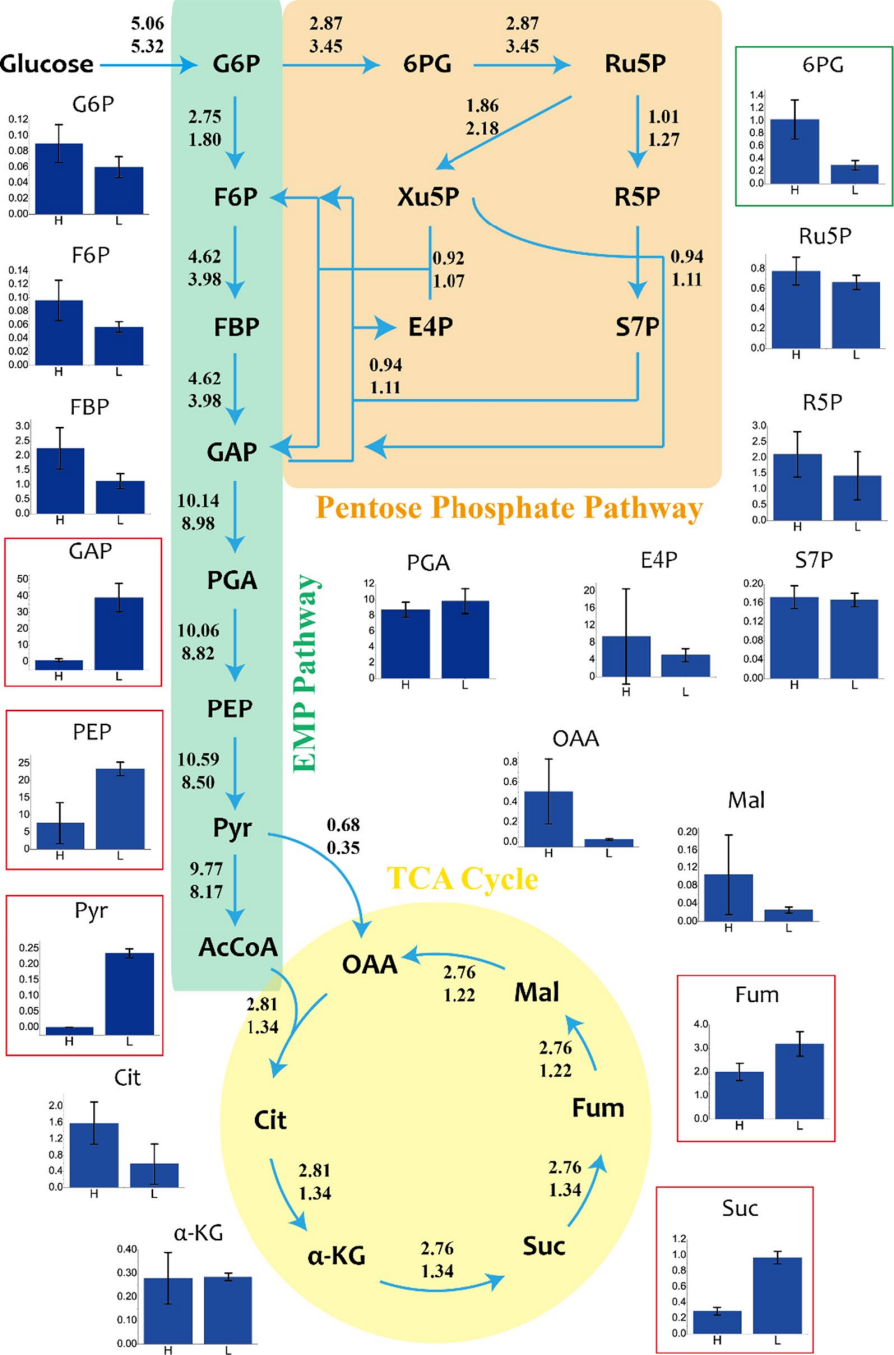
Metabolite pools and flux distribution between Batch H (left columns and lower numbers) and Batch L (right columns and upper numbers). The bar graphs with frame indicate the significant difference (*P* < 0.05) between two batches. Red frames indicate higher concentration in Batch L while green one indicating lower concentration in Batch L. The units on the y-axes are μmol/gDCW

In terms of metabolite pools, *B. subtilis* showed obvious differences between two batches (Fig. 6). In the Batch H, the pool sizes of the metabolites in upstream of EMP pathway were larger than that in Batch L. However, in the downstream of EMP pathway, the pool sizes of most metabolites were significantly smaller in Batch H compared with Batch L. It was interesting because the EMP pathway is divided into two parts based upon ATP’s production. It was reasonable to speculate that these differences in EMP metabolite pool sizes were generated by energy metabolism.

When it came to TCA cycle, the metabolite pool sizes were larger in the Batch H, except for succinate and fumarate (Fig. 6). This phenomenon was agreed with the trends of organic acids those were secreted into culture medium (Fig. 3). The concentration of 6-PG, the metabolite connecting PP and EMP pathway, was found significantly larger in Batch H and that may be the consequence of larger flux through PP pathway in Batch H.

**Fig. 7:**
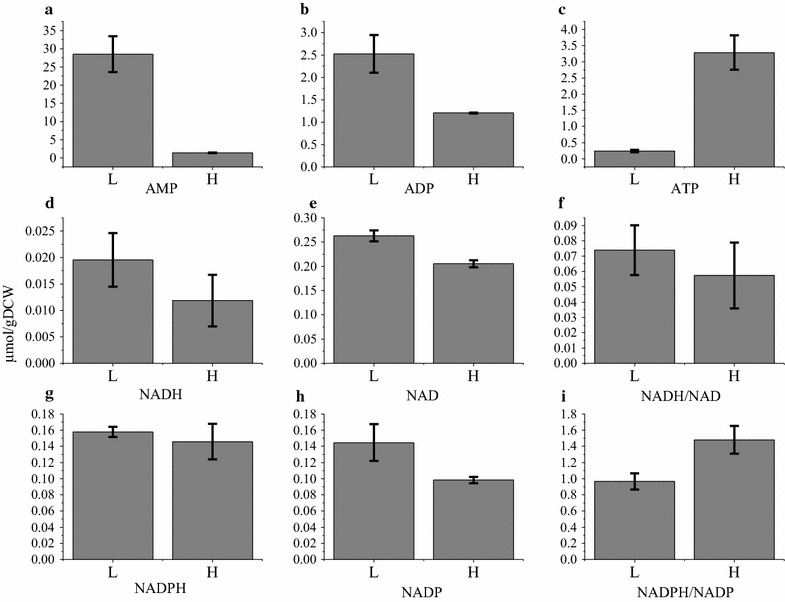
Pool sizes of **a** AMP, **b** ADP, **c** ATP, **d** NADH, **e** NAD, **g** NADPH and **h** NADP. Left bars represent Batch L, right bars represent Batch H. Values in **f** and **i** represent ratio of NADH/NAD and NADPH/NADP, respectively

From the metabolic pool sizes analysis, many details were found to support the MFA analysis (Figs. 4, 5). Significant difference was found on the pool size of pyruvate. In Batch H, intracellular pyruvate was undetectable indicating a very low concentration, while intracellular pyruvate concentration in Batch L was 0.02357 μmol/gDCW. As pyruvate is the metabolite linking EMP pathway and TCA cycle, it was reasonable that higher pyruvate pool size in Batch L increased the flux through TCA cycle (Figs. 4, 6). More than ten times of ATP pool size in Batch H than that obtained in Batch L verified the calculation in MFA (Figs. 5, 7). Higher ratio of NADH/NAD^+^ and lower ratio of NADPH/NADP^+^ in Batch L was also agreed with the metabolic fluxes distributions and calculations (Figs. 5, 7). MFA results represented how the metabolites changing and metabolomics results showed the status of different metabolites. Integrating these results can show a deeper insight into the cell metabolic regulation. However, how can the oxygen tension influence the metabolic reactions and metabolite pool sizes? So transcriptomics analysis was performed in the following experiment.

### Gene expression profiling

**Fig. 8:**
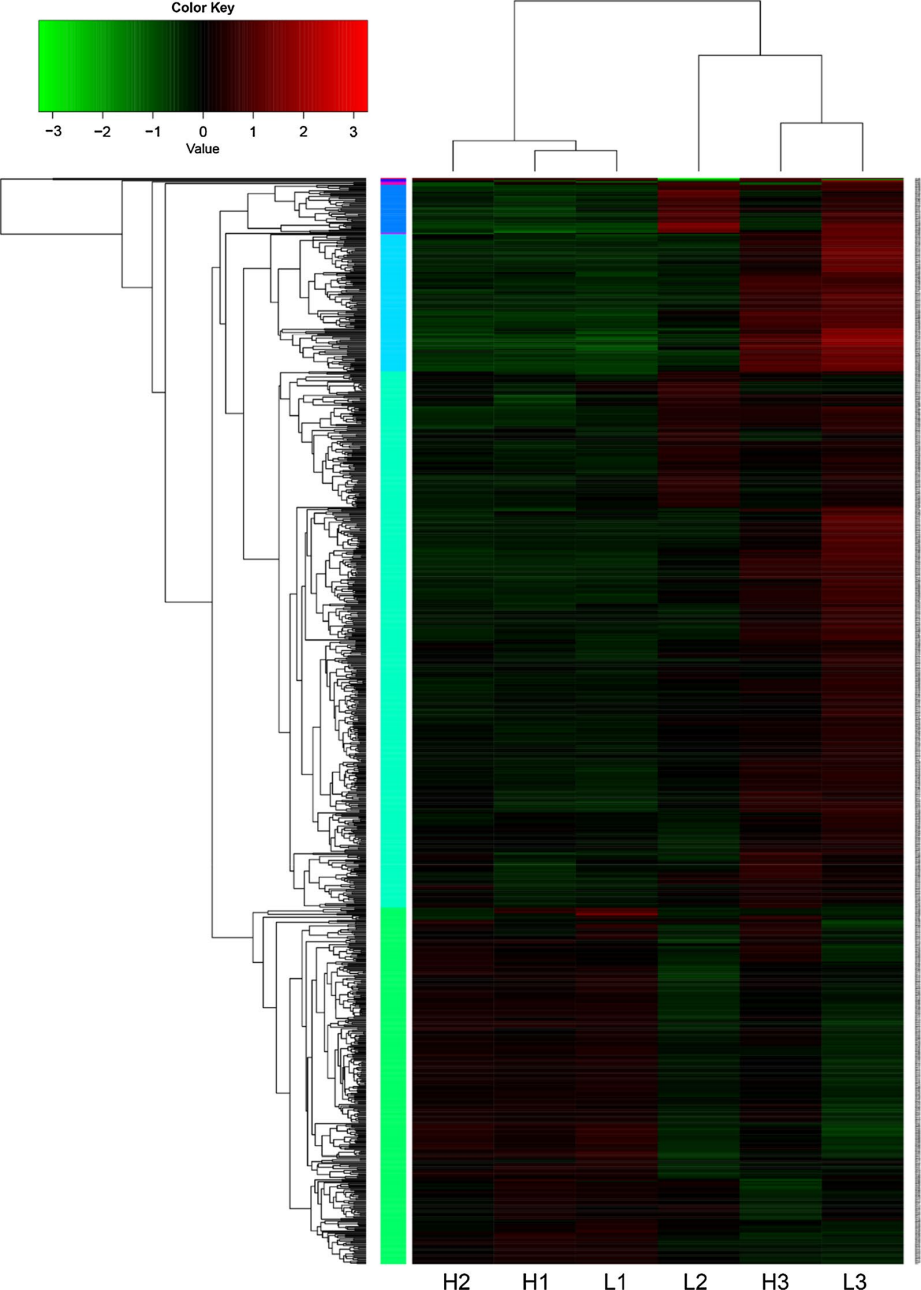
Clustering of all genes among six sample points. H1, H2 and H3 represent three samples of Batch H. L1, L2 and L3 represent three samples of Batch L. Color of each lattice represents expression of one gene in one sample

A time series of a global transcription analysis was carried out for these two batches. Different regulation strategies of genes while facing different oxygen availability conditions can be discovered by clustering the expression of all genes among the six sample points (Fig. 2). The distance between samples was evaluated by Spearman correlation coefficient, while between genes was done by Pearson correlation coefficient while clustering method used was hcluster. The result of the clustering is shown in Fig. 8. It indicates that gene expression profiling between the exponential growth phases of two batches (H1 and L1) are most similar among these six points, the situation of transition phase under high oxygen availability (H2) is similar to the one in exponential growth phase and the one in transition phase with low oxygen availability (L2) is similar to the situation of stable phase (H3 and L3). Although the DO tension was set over critical DO level during the whole process of Batch H, the gene expression profiling of H3 is similar to L3 rather than to H1 or H2.

**Fig. 9:**
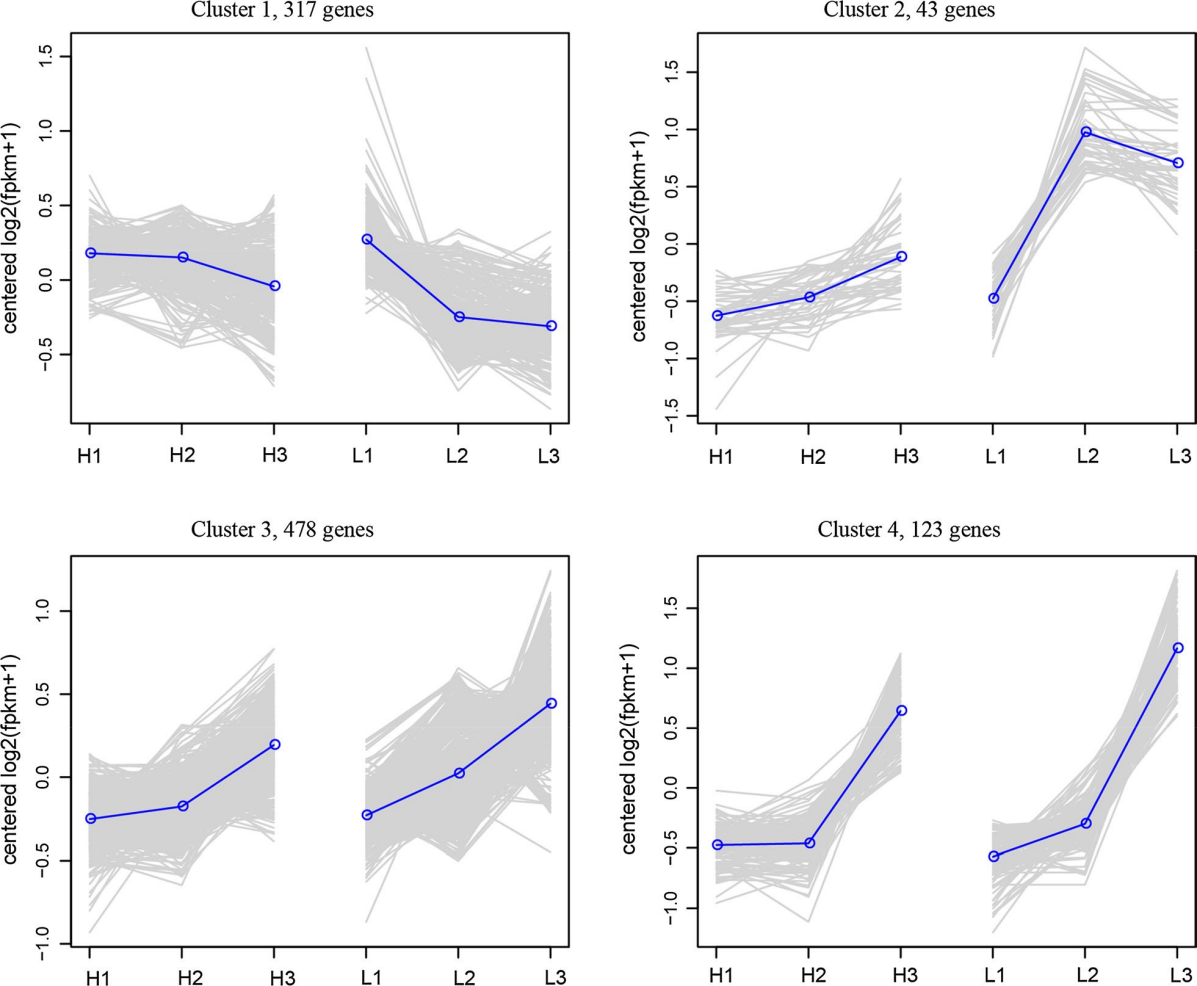
Express trends of main clusters of genes. H1, H2 and H3 represent three sampling time points of Batch H. L1, L2 and L3 represent three sampling time points of Batch L

**Fig. 10:**
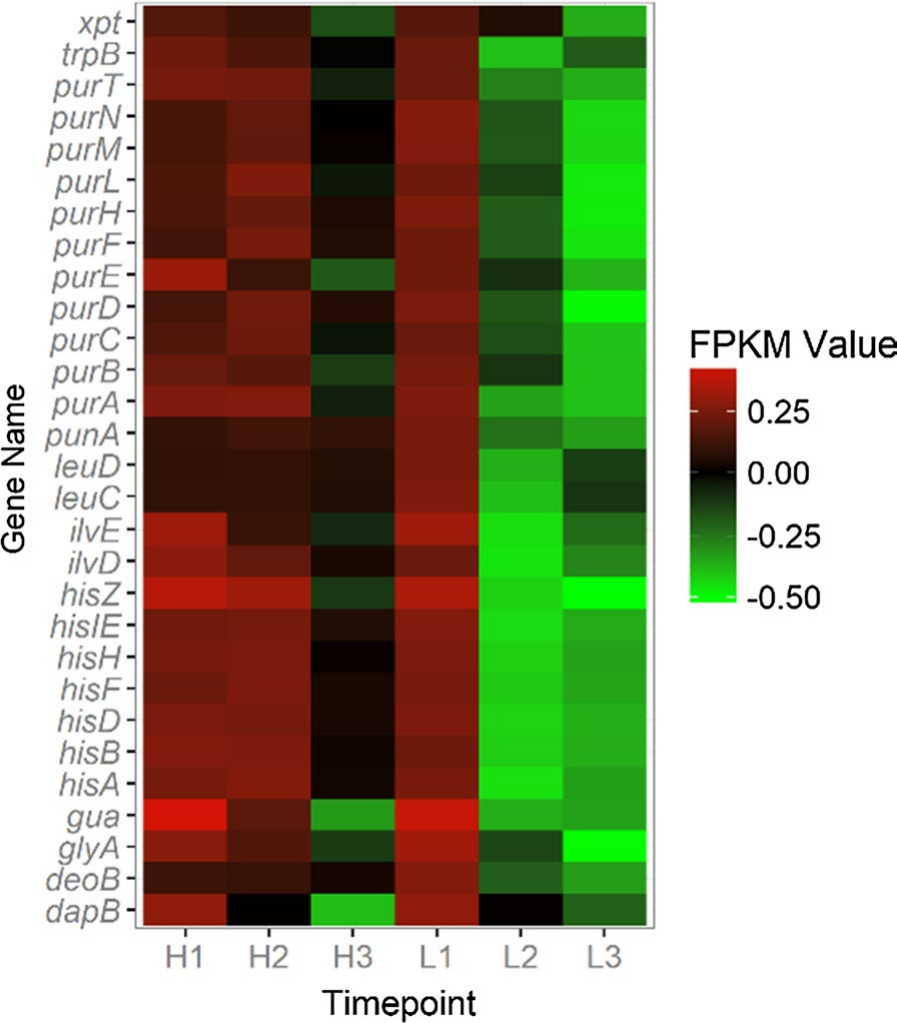
Transcription profiling of genes that related to histidine metabolism and purine metabolism. H1, H2 and H3 represent three sampling time points of Batch H. L1, L2 and L3 represent three sampling time points of Batch L. Color of each lattice represents expression of one gene in one sample

**Fig. 11:**
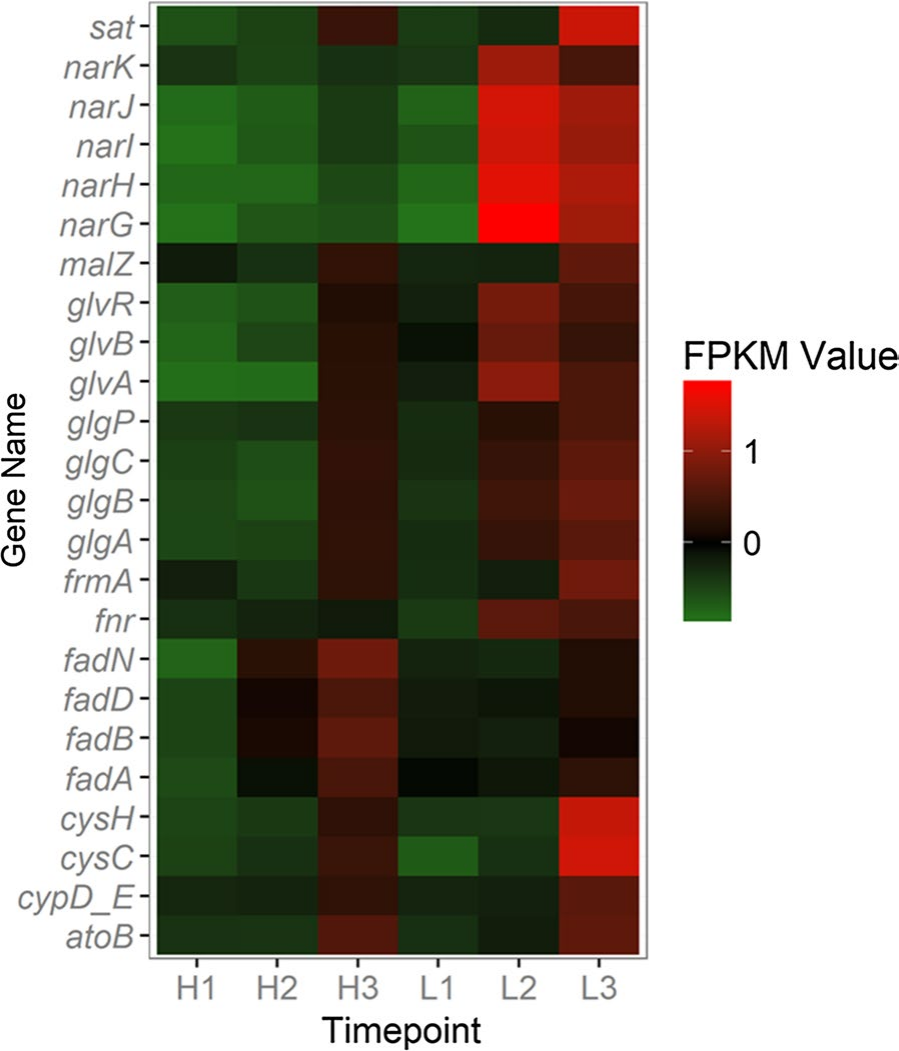
Transcription profiling of genes that related to fatty acid degradation, starch and sucrose metabolism, sulfur metabolism and nitrogen metabolism. H1, H2 and H3 represent three sampling time points of Batch H. L1, L2 and L3 represent three sampling time points of Batch L. Color of each lattice represents expression of one gene in one sample

In the clustering of all genes, it is obvious that all genes were divided into 4 main clusters (Fig. 9). The genes that belong to the second, third and fourth cluster were all showing a trend that their expressions were increasing during the process. The genes that involved in the metabolic network were selected and heat maps were made. It was observed that when the level of DO decreased, genes that involved in the histidine formation from phosphoribosyl pyrophosphate (PRPP) and IMP formation from PRPP were significantly inhibited (Fig. 10). It should be noticed that these routes are included in the amino acid metabolism and purine metabolism which is the pathway that provides precursors to produce riboflavin. On the contrary, the genes involved in energy metabolism (starch and sucrose metabolism, fatty acid degradation and fructose and mannose metabolism) and nitrogen metabolism were activated under low DO concentration (Fig. 11). From this gene expression profiling analysis based on time course, a set of genes (*pur*) were highlighted as they encoded enzymes that catalyze pathways for riboflavin generation but were inhibited in the later phase of fermentation process. These genes may be potential genetically modified targets to improve the riboflavin biosynthesis.

### Differential expression analysis of genes

To study the regulatory mechanism of *B. subtilis* grown under low dissolved oxygen tension stress, the differential expression analysis of genes between the same phases under two conditions was performed. It was found that there were 224 and 169 differential expression between two conditions in the second and third phase respectively; and 53 genes were overlapped. Interactive pathways explorer 2 (http://pathways.embl.de/iPath2.cgi#) was used to show the results of the analysis (Figures shown in Additional file 2). It is obvious that the reaction from pyruvate to acetoin (*alsS*) was up-regulated under low oxygen availability. This can be explained that this reaction was proved to be propitious to stabilize the ratio of NAD^+^/NADH and maintain the redox balance [18]. In addition, the genes that related to the reactions that generate cytochrome bd, from maltose to oxaloacetate and from polysaccharide like starch and sucrose to glucose were also up-regulated. Besides, ResD–ResE, one two-component regulatory factor which has been proved to be responsible for the gene regulation to adapt oxygen tension shifting in *B. subtilis* [19, 20], was up-regulated significantly in Batch L based on transcriptomic analysis. It was reasonable to suppose that this two-component regulatory factor received the signal of low DO tension and regulated the gene expression globally in cells tested here.

**Fig. 12:**
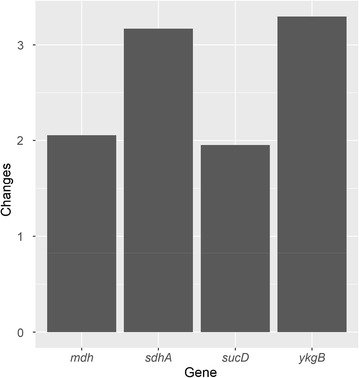
The fold changes in four genes expression in Batch H, compared with Batch L

To explain the results gotten from MFA and metabolomics, the genes involved in central carbon metabolism were further studied. Four genes (*mdh*, *sdhA*, *ykgB* and *sucD*) were picked because obvious differences lied between the two batches. To verify these differences showed by RNA-seq, qRT-PCR was performed and a consistent result was gained (Fig. 12). The gene *ykgB* which encodes 6-phosphogluconolactonase is a key gene that linking EMP pathway and PP pathway. Lower expression of *ykgB* (0.3035 times) may be responsible for lower flux through PP pathway in Batch L than that in Batch H.

From transcriptomics analysis, global gene expression profiling was performed and the genes involved in purine metabolism were picked out as potential manipulating targets to optimize riboflavin fermentation. Activated ResD–ResE system was supposed to be the sensor of DO tension and regulator of relative genes for the adaptation of DO shift. Gene *alsS* and *ykgB* were thought to be the key genes that redistribute the fluxes through central carbon metabolism.

## Discussion

### Central carbon metabolism

Based on the result of ^13^C MFA, the relative flux through PP pathway was significantly larger in Batch H than Batch L, while the situation was vice versa in EMP pathway and TCA cycle (Fig. 5). Various studies proved that, gene *zwf* is the key gene which can regulate the flux through PP pathway [17, 21]. The manners that regulate the flux shift between PP and EMP pathway are different among organisms and aren’t controlled by the demand of NADPH or pentose [17, 22]. Various signal transduction pathway, in response to different factors in organisms, may affect different genes. It is speculated that when DO concentration decreases, a number of regulatory factors are activated by ResE–ResD leading to a repressed expression of *ykgB*. Beside the latter phenomenon, cellular physiological status can also affect the gene expression, i.e. higher NADPH/NADP^+^ ratio repressed expression of *zwf* in Batch H. In our study, the gene *ykgB* which encodes 6-phosphogluconolactonase catalyzing the formation of 6-phosphogluconate from 6-phosphogluconolactone was expressed higher in Batch H than Batch L and this may be responsible for the increased flux through PP pathway in Batch H. Obvious difference of expression in *zwf* between two conditions was not observed in this case. The transcription of *zwf* in Batch H might be suppressed by higher NADPH/NADP^+^ ratio. Because of the larger flux through PP pathway, it is not surprising to find out the pool sizes of main metabolites in PP pathway were larger in Batch H. PP pathway is the main source of ribulose 5-phosphate which is one of the precursor of riboflavin, and links with purine metabolism responsible for producing GTP, one another precursor of riboflavin, by PRPP. Besides, PP pathway is a major source of reducing power and metabolic intermediates for cell growth [23]. To summarize, the rise in flux through PP pathway would be attributed as the main reason for higher biomass and riboflavin production.

Higher flux through EMP pathway was observed in Batch L compared with Batch H. It was hypothesized that due to lack of oxygen, NADH/NAD^+^ ratio (Fig. 7) was increased which may suppress the conversion from pyruvate to acetyl-CoA [24]. Hence, pyruvate was difficult to be consumed and subsequently led to accumulation of EMP downstream metabolites (Fig. 5). The genes that encode acetolactate synthase were significantly up-regulated both at the second and third time point in Batch L compared with Batch H. This reaction converts pyruvate to acetoin which was proved to play an important role in maintaining the NAD^+^/NADH balance [18]. The vast amount of pyruvate (both extracellular and intracellular) and absence of electron acceptor in Batch L may be the main reason for this stronger expression.

It was surprised to find out that the flux through TCA cycle was higher in Batch L than Batch H, because TCA cycle is a redox process that relies on oxygen. The only reasonable interpretation was the large accumulation of pyruvate that links the EMP pathway and TCA cycle. The high driving force enlarged the flux through TCA cycle, but the NADH produced by this process was accumulated instead of oxidizing to NAD^+^ by oxygen. Even so, the accumulated NADH can be converted into NAD^+^ by substrate phosphorylation and that eventually supported the higher flux through EMP pathway in Batch L.

### Purine metabolism

Purine metabolism is important for riboflavin production, because it provides GTP for formation of riboflavin. Comparing the expression of genes that were involved in purine metabolism at all three time points in between Batch H and Batch L; no significant difference was found between the same time points of two batches. However, obvious expression changing trend of genes in purine metabolism (Fig. 10) was found while clustering the whole genes. In two batches, one similar trend was showed that most of the *pur* genes expression decreased along with the bioprocess both in Batch H and Batch L, although with different degree. This trend indicated that the purine metabolism may be affected by glucose concentration and oxygen availability as those genes were down-regulated more significantly under low DO condition (Fig. 10). And this may be the potential target manipulation for explaining the productivity loss of riboflavin during later phase.

### How to compensate lack of electron acceptor

**Fig. 13:**
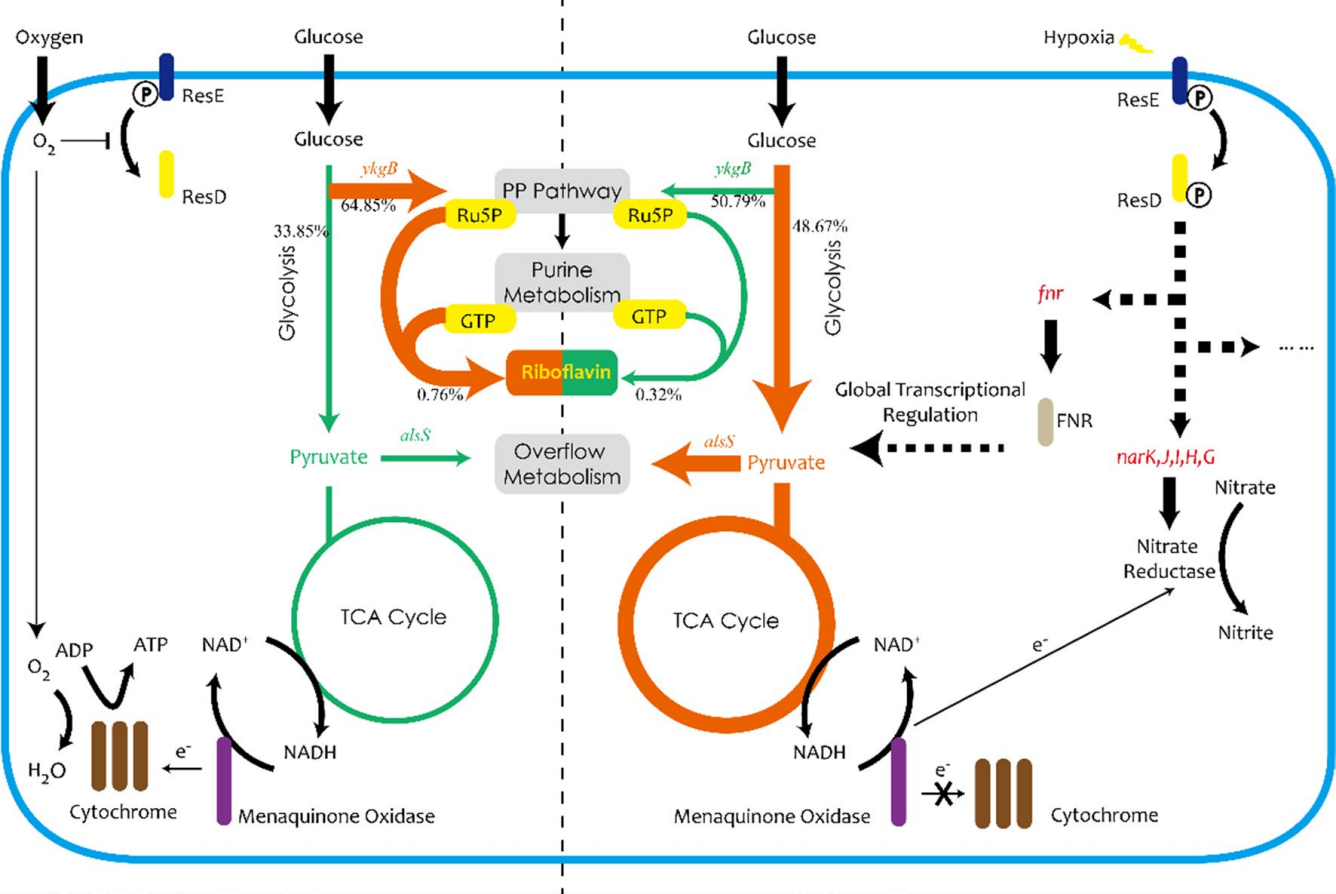
Speculated regulatory mechanism of dissolved oxygen tension in *B. subtilis* cells. When the DO concentration decreased (left) ResE can activate ResD, a regulatory protein that can regulate the expression of genes *fnr*, *narK, J, I, H, G* and so on. Global regulator FNR was speculated to be responsible for the down-regulated *ykgB* and up-regulated *alsS*, which lead to decreased flux through PP pathway, lower riboflavin production and increased flux through glycolysis and overflow metabolism. Nitrate accepted more electrons as genes *narK, J, I, H, G* were activated. When the DO concentration was high enough (right) ResE will dephosphorylate ResD and enough oxygen molecules can maintain the normal operation of electron transport chain

Higher concentrations of NADH and NADPH along with lower ATP were the immediate consequences that emerged from Batch L. To compensate this unbalance, *B. subtilis* sought the alternative electron acceptor, like nitrate and sulfate. From the comparison of global gene expression profiling, it was obvious that the genes encode nitrate reductase (*narK, J, I, H, G*) were highly up regulated. This agreed with Vetter and Schlievert [19] that *B. subtilis* used nitrogen as a final electron acceptor while growing anaerobically. Besides, the expression of two-component system regulator ResD–ResE that can regulate nitrate reductases was also up-regulated significantly in Batch L, indicating that some of the response reactions were regulated by this system. This two-component system has been proved to be implicated in the global regulation of aerobic and anaerobic respiration of *B. subtilis* and can sense the extracellular oxygen concentration and regulate genes like nar*K, J, I, H, G* and *fnr* [20, 25–27] (Fig. 11). Gene *fnr* encodes a global transcriptional regulator FNR which can trigger several transcriptional responses to adapt low oxygen tension. In some studies, FNR showed ability to regulate the central carbon metabolism [28, 29]. Therefore, it was reasonable to speculate that FNR performed global transcriptional regulation function and regulated the genes in central carbon metabolism like *ykgB*, *alsS*, *sucC*, *sdhA* and *mdh* while *B. subtilis* grew under low DO concentration. ResE is a sensor kinase which has histidine kinase activity and phosphatase activity. ResE can phosphorylate ResD as a histidine kinase and dephosphorylate ResD only when the oxygen tension was low. In summary, ResE receive the signal of oxygen tension then deliver to ResD, making it phosphorylated or dephosphorylated. These different status of ResD subsequently lead to different gene expression pattern and metabolic flux distribution (Fig. 13).

### Future prospects

Current study focuses on momentous effect of DO concentration upon riboflavin production. Carbon flux distributions, intracellular metabolite pool size and gene expression profile aided in obtaining major conclusions for this effect. Furthermore, covering of different supporting omics for the conducted study validates the consideration of above stated parameters. However, there lie certain future prospects that can further unveil extent of such promising DO concentration effect for enhancing biosynthesis. Specifically, if pur-related genes, *ykgB* overexpression or *alsS* knocking can improve the riboflavin production under low DO condition? If expressions of *ykgB* or *alsS* could be regulated by FNR regulator. If providing more nitrate can reduce the NADH/NAD^+^ ratio, improve ATP generation and riboflavin production? If co-feeding strategy on using different carbon source as substrates could be applied to alleviate the formation of by-products? [22]. All these hypotheses on exploration can open new avenues for effective resource utilization in biosynthesis.

## Conclusions

In this paper, integration of ^13^C metabolic flux analysis, metabolomics and transcriptomics unveils the responses of *B. subtilis* towards low oxygen tension. ResDE system was verified to be the signal transduction between DO concentration and gene expression by transcriptomics. In addition, the shift in flux from PP to EMP pathway is considered as the main reason of declined riboflavin production under low oxygen availability due to less precursors and reducing power supply as per metabolomics and transcriptomics. The genes involved in purine metabolism would be regarded as genetically possible manipulated targets to maintain high riboflavin production in the later phase of fermentation.

## Additional files

**Additional file 1.** Mass isotopomer distributions (MID) of intracellular free amino acids.

**Additional file 2.** Results of iPath analysis.
