# Supplementary material for: Mixomics analysis of Bacillus subtilis: effect of oxygen availability on riboflavin production
Source: Microb Cell Fact. 2017 Sep 12;16:150. doi: 10.1186/s12934-017-0764-z (PMC5596917; doi:10.1186/s12934-017-0764-z)
Supplement: Supplementary file 1 — Additional file 1. Mass isotopomer distributions (MID) of intracellular free amino acids. [file 12934_2017_764_MOESM1_ESM.docx]

Additional file 1

Mass isotopomer distributions (MID) of intracellular free amino acids (Batch L)

| Amino acid MID | Measured | Estimated | SRES |
| --- | --- | --- | --- |
| Ala-15 | 0.2640  0.2280  0.2130  0.2950 | 0.2749  0.2295  0.1988  0.2932 | 1.1925  0.0222  2.0081  0.0328 |
| Ala-159 | 0.3160  0.3370  0.3470 | 0.3228  0.3315  0.3459 | 0.4674  0.3038  0.0122 |
| Asp-57 | \| 0.1640 \| \| --- \| \| 0.2230 \| \| 0.2040 \| \| 0.2160 \| \| 0.1930 \| | 0.1660  0.2303  0.1957  0.2182  0.1884 | 0.0385  0.5339  0.6883  0.0480  0.2085 |
| Asp-85 | 0.1910  0.2900  0.2760  0.2430 | 0.2092  0.2805  0.2786  0.2351 | 3.3137  0.9068  0.0686  0.6268 |
| Gly-57 | 0.4110  0.1570  0.4320 | 0.4273  0.1646  0.4121 | 2.6456  0.5834  3.9604 |
| Gly-85 | 0.5050  0.4950 | 0.5059  0.4940 | 0.0087  0.0092 |
| Glu-57 | 0.0990  0.1430  0.2370  0.2610  0.1550  0.1050 | 0.0858  0.1456  0.2453  0.2579  0.1543  0.1012 | 1.7399  0.0697  0.6953  0.0971  0.0050  0.1463 |
| Glu-85 | 0.1460  0.1550  0.3580  0.1660  0.1750 | 0.1518  0.1570  0.3575  0.1655  0.1695 | 0.3354  0.0387  0.0025  0.0023  0.3003 |
| Lys-57 | 0.0640  0.1210  0.1880  0.2260  0.1980  0.1360  0.0670 | 0.0534  0.1289  0.1962  0.2142  0.2001  0.1374  0.0649 | 1.1285  0.6207  0.6704  1.4006  0.0440  0.0200  0.0427 |
| Lys-159 | 0.0620  0.1630  0.2580  0.2650  0.1710  0.0810 | 0.0676  0.1600  0.2555  0.2655  0.1744  0.0814 | 0.3121  0.0894  0.0641  0.0021  0.1173  0.0014 |
| Phe-57 | 0.0740  0.0690  0.1180  0.1270  0.1220  0.1290  0.1140  0.1110  0.0590  0.0770 | 0.0718  0.0601  0.1125  0.1260  0.1278  0.1408  0.1122  0.1102  0.0512  0.0733 | 0.0504  0.7890  0.3003  0.0094  0.3362  1.3878  0.0335  0.0069  0.6018  0.1398 |
| Phe-85 | 0.0810  0.0550  0.1850  0.0730  0.1820  0.0780  0.1850  0.0690  0.0920 | 0.0844  0.0652  0.1921  0.0794  0.1798  0.0722  0.1784  0.0637  0.0861 | 0.1181  1.0395  0.5101  0.4154  0.0502  0.3310  0.4365  0.2783  0.3429 |
| Leu-159 | 0.0630  0.1540  0.2670  0.2760  0.1760  0.0640 | 0.0481  0.1535  0.2660  0.2805  0.1754  0.0627 | 2.2073  0.0030  0.0095  0.1989  0.0039  0.0178 |
| Pro-159 | 0.1680  0.1530  0.3580  0.1590  0.1620 | 0.1516  0.1567  0.3570  0.1653  0.1693 | 2.6994  0.1397  0.0104  0.3938  0.5289 |
| emp1 | 5.6600 | 5.6597 | 0.0000 |
| rib | 0.0380 | 0.0179 | 4.0242 |
| biomass | 0.0350 | 0.0436 | 0.7479 |

SSR = 42.8, expected SSR = [15.3 44.5]

Mass isotopomer distributions (MID) of intracellular free amino acids (Batch H)

| Amino acid MID | measured | estimated | SRES |
| --- | --- | --- | --- |
| Asp-57 | \| 0.1623 \| \| --- \| \| 0.2461 \| \| 0.1340 \| \| 0.2534 \| \| 0.2042 \| | 0.1629  0.2604  0.1351  0.2371  0.2037 | 0.0032  1.8595  0.0109  2.4156  0.0024 |
| Asp-85 | 0.1940  0.2920  0.2690  0.2450 | 0.2002  0.2869  0.2687  0.2463 | 0.3811  0.2573  0.0011  0.0159 |
| Gly-57 | 0.4160  0.1670  0.4170 | 0.4208  0.1677  0.4118 | 0.2338  0.0053  0.2745 |
| Gly-85 | 0.5070  0.4930 | 0.5055  0.4945 | 0.0211  0.0221 |
| Glu-57 | 0.0860  0.1480  0.2360  0.2690  0.1480  0.1130 | 0.0870  0.1468  0.2391  0.2667  0.1489  0.1116 | 0.0097  0.0154  0.0961  0.0545  0.0082  0.0197 |
| Glu-85 | 0.1802  0.1055  0.3989  0.1022  0.2132 | 0.1876  0.0952  0.4033  0.0993  0.2040 | 0.4463  0.8745  0.1588  0.0696  0.7020 |
| His-159 | 0.2012  0.2406  0.0586  0.0617  0.2154  0.2224 | 0.2005  0.2475  0.0550  0.0548  0.2201  0.2130 | 0.0049  0.4570  0.1268  0.4693  0.2207  0.8779 |
| Phe-57 | 0.0780  0.0520  0.1070  0.1260  0.1280  0.1330  0.1350  0.1100  0.0510  0.0800 | 0.0692  0.0545  0.1101  0.1304  0.1303  0.1410  0.1214  0.1126  0.0499  0.0737 | 0.7695  0.0615  0.0991  0.1941  0.0549  0.6459  1.8632  0.0655  0.0126  0.3934 |
| Phe-85 | 0.0891  0.0480  0.1942  0.0721  0.1982  0.0741  0.1922  0.0400  0.0921 | 0.0870  0.0541  0.1946  0.0785  0.1941  0.0757  0.1881  0.0550  0.0911 | 0.0456  0.3653  0.0014  0.4131  0.1701  0.0265  0.1681  2.2238  0.0095 |
| Leu-57 | 0.0594  0.0950  0.2287  0.2020  0.2426  0.1050  0.0673 | 0.0603  0.0914  0.2331  0.1977  0.2443  0.1003  0.0695 | 0.0088  0.1342  0.1949  0.1908  0.0296  0.2161  0.0491 |
| Leu-159 | 0.0830  0.1580  0.2570  0.2630  0.1530  0.0860 | 0.0654  0.1541  0.2538  0.2657  0.1672  0.0791 | 3.098  0.1517  0.1005  0.0753  2.0183  0.4731 |
| emp1 | 5.3200 | 5.3200 | 0.0000 |
| rib | 0.0540 | 0.0402 | 1.9165 |
| biomass | 0.0920 | 0.0977 | 0.3253 |

SSR = 26.8, expected SSR = [8.9 32.9]
