# Supplementary material for: Mixomics analysis of Bacillus subtilis: effect of oxygen availability on riboflavin production
Source: Microb Cell Fact. 2017 Sep 12;16:150. doi: 10.1186/s12934-017-0764-z (PMC5596917; doi:10.1186/s12934-017-0764-z)
Supplement: Supplementary file 2 — Additional file 2. Results of iPath analysis. [file 12934_2017_764_MOESM2_ESM.pdf]

Additional file 2

Results of iPath analysis

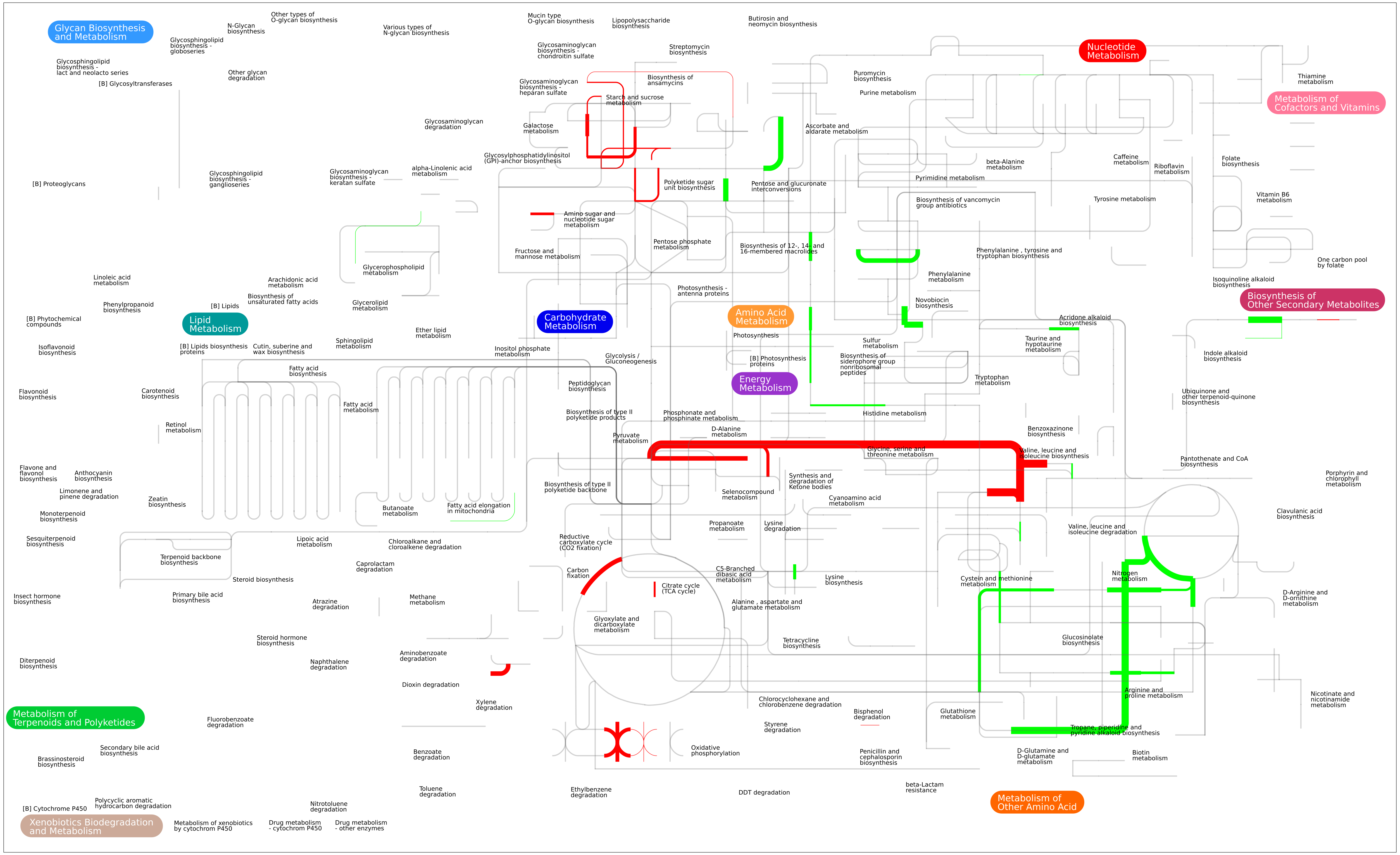

Differentially expressed genes involved in metabolic pathways (L2 vs H2).
Reactions that highlighted in red represent upregulation of corresponding genes under low DO mode and that highlighted in green represent downregulation of corresponding genes under low DO mode.
Thicker the line is represent greater difference in gene expression.

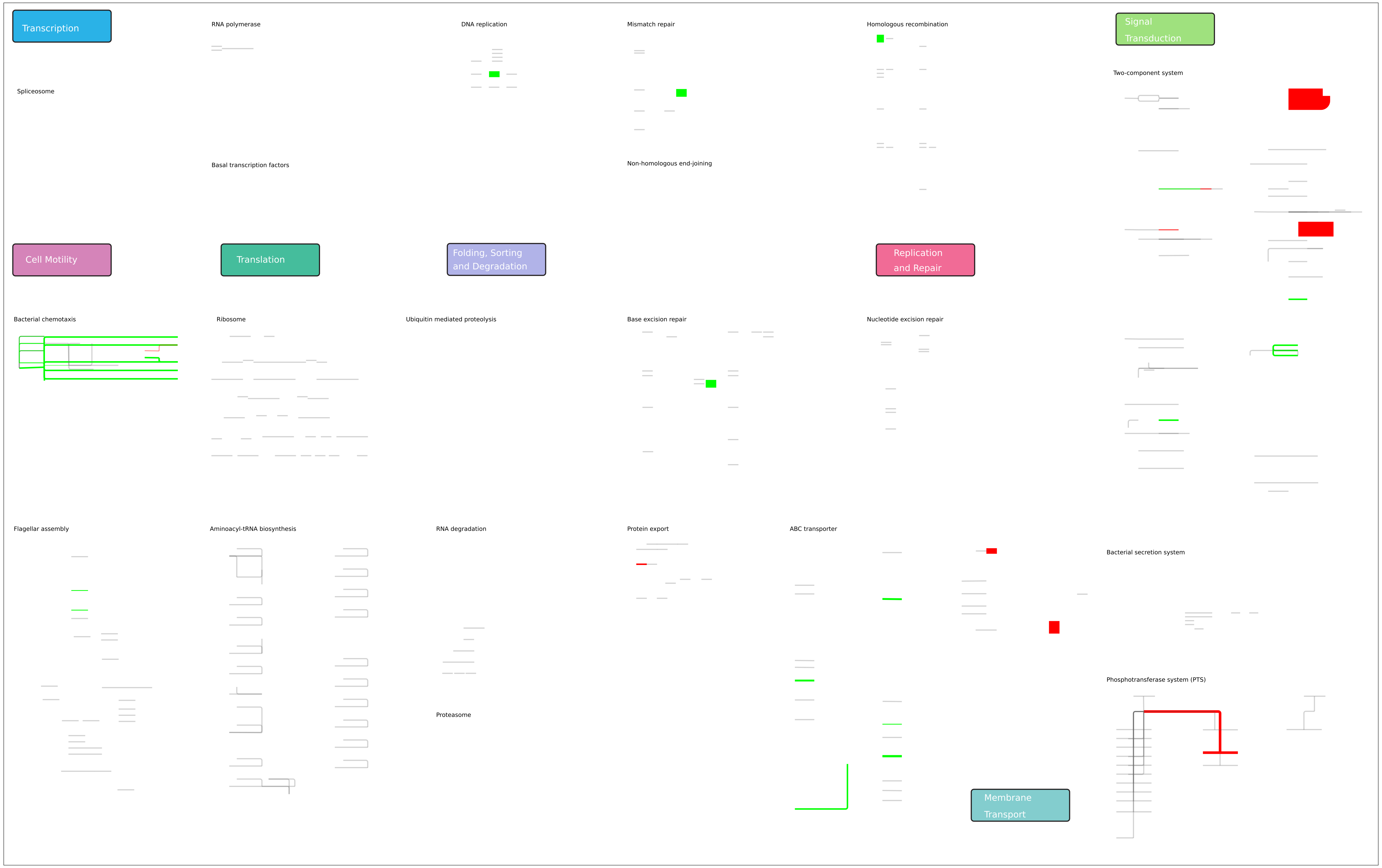

Differentially expressed genes involved in regulatory pathways (L2 vs H2).

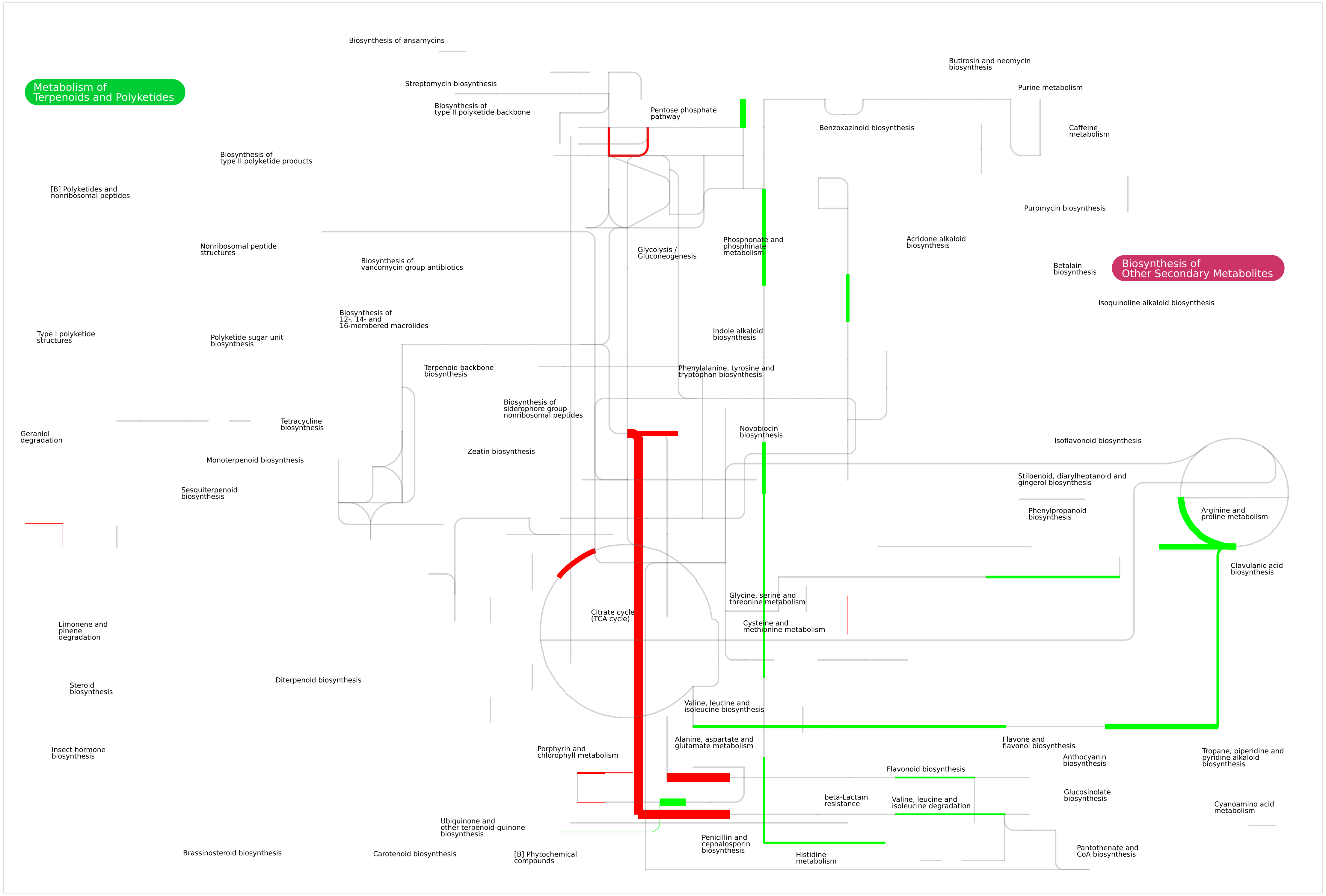

Differentially expressed genes involved in metabolites biosynthesis (L2 vs H2).

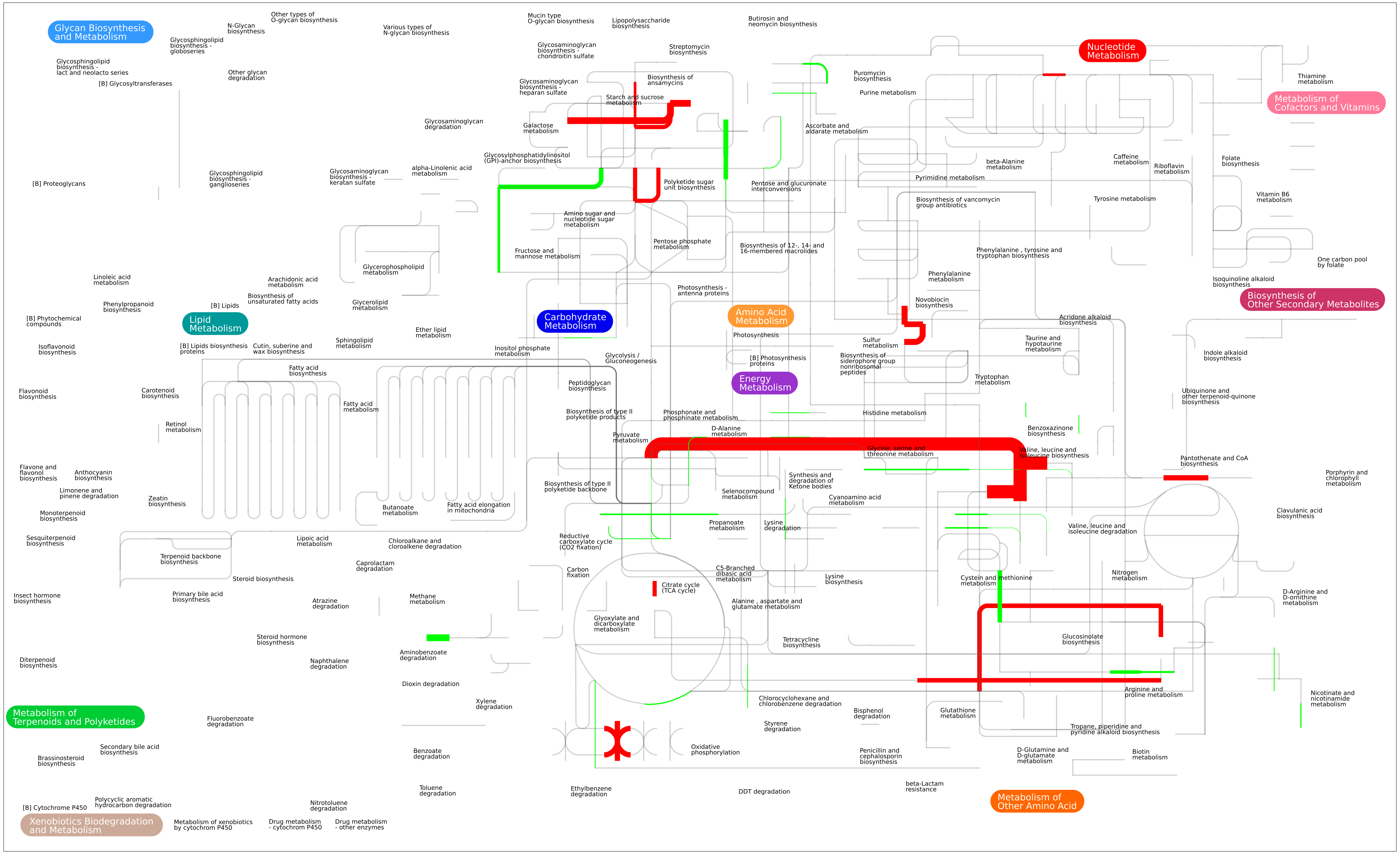

Differentially expressed genes involved in metabolic pathways (L3 vs H3).

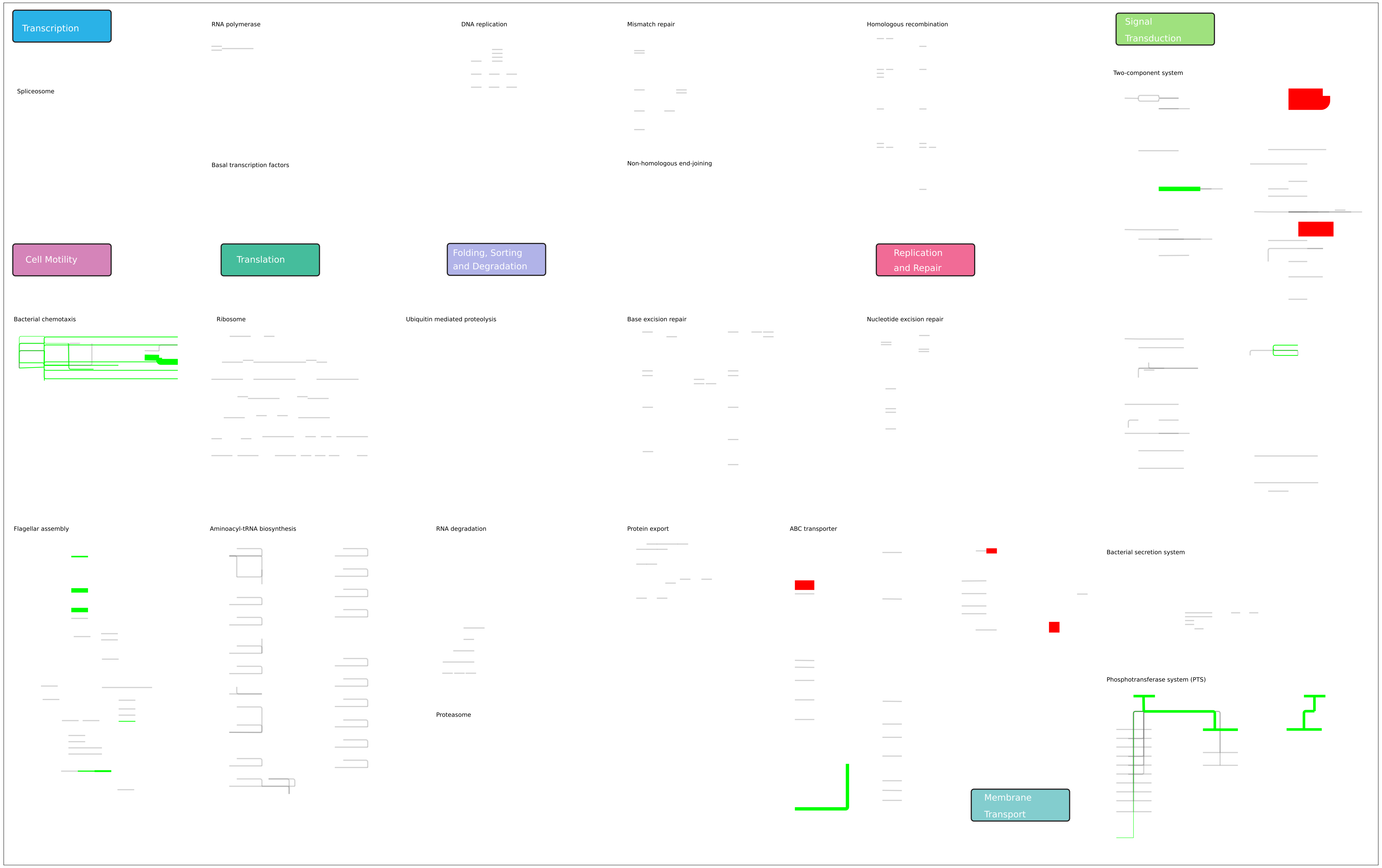

Differentially expressed genes involved in regulatory pathways (L3 vs H3).

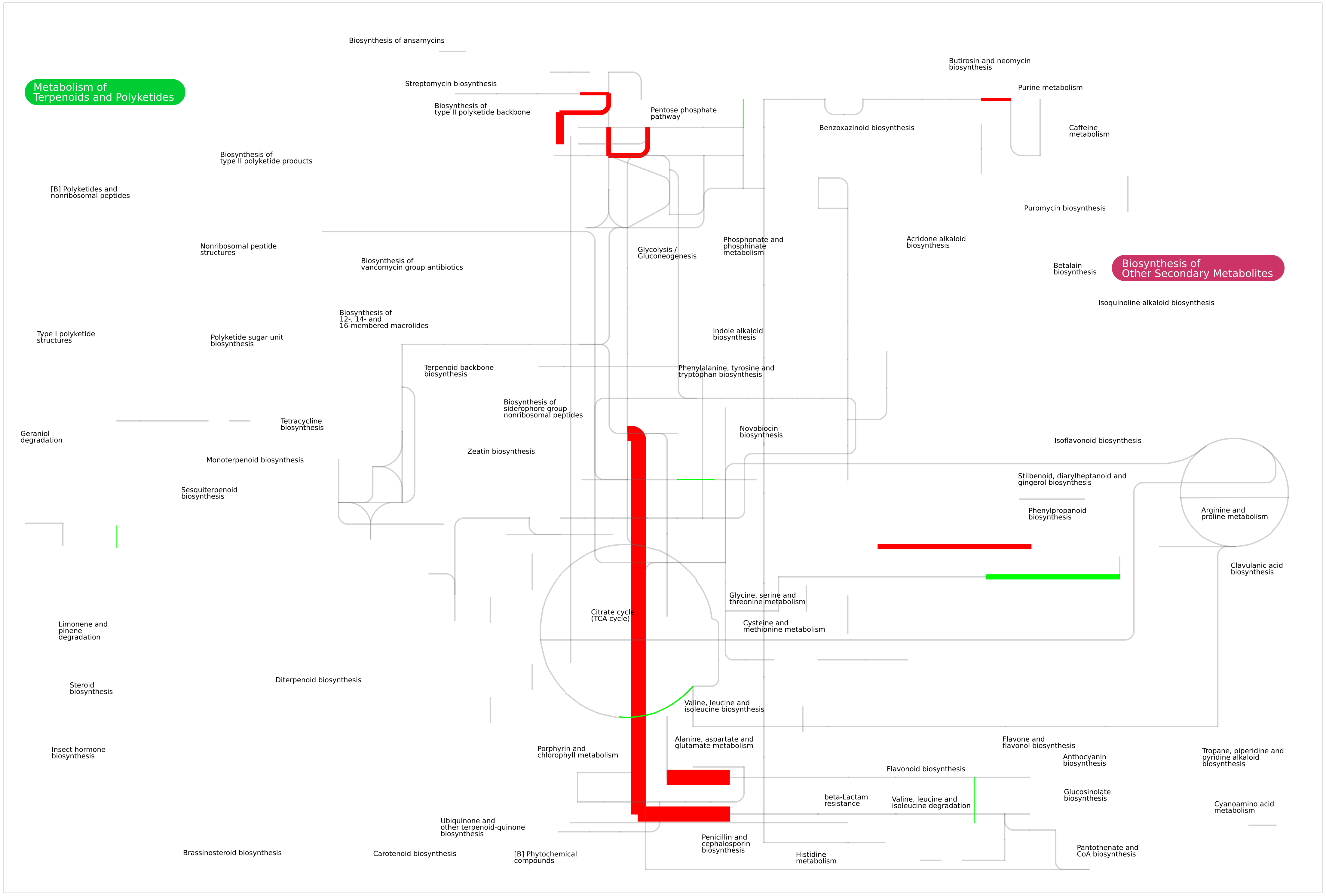

Differentially expressed genes involved in metabolites biosynthesis (L3 vs H3).
